# Predictors of culture status in patients with persistent smear-positive pulmonary tuberculosis at two months of treatment

**DOI:** 10.1186/s12879-025-12173-x

**Published:** 2026-01-23

**Authors:** Junais Koleri, Faraj S. Howady, Jay P. N. Singh, Sara Al Balushi, Muna Al Maslamani

**Affiliations:** 1 Division of Infectious Diseases, Hamad Medical Corporation, PO Box 3050, Doha, Qatar; 2 Laboratory Medicine, Hamad Medical Corporation, Doha, Qatar

**Keywords:** Pulmonary TB, Smear positivity, Culture status, Respiratory isolation, TB infectivity

## Abstract

**Introduction:**

In pulmonary tuberculosis (TB), smear positivity usually declines with effective treatment, but the time to non-infectiousness varies, creating uncertainty about the optimal duration of isolation. The Centers for Disease Control and Prevention (CDC) 2005 guidelines allow discharge before smear conversion to home isolation (restricted to healthcare visits until smear negativity) if no vulnerable household contacts are present, whereas hospitalized patients are advised to remain under airborne precautions until they have three consecutive negative smears. The practice in Qatar is to keep sputum smear positive TB patients in isolation facilities until smear negativity is achieved. Relying solely on smear conversion as a marker of non-infectiousness is problematic, as persistent smear positivity may reflect nonviable bacilli, or in some cases non-tuberculous mycobacteria, rather than ongoing transmission risk. This study evaluates the culture status of patients who remained smear-positive after two months of therapy to determine bacillary viability and reassess the validity of smear-based isolation practices.

**Aim:**

This study aimed to determine the proportion of culture-positive cases among pulmonary tuberculosis patients remaining smear-positive at two months of treatment and identify factors predictive of culture-negative status to support earlier isolation discontinuation.

**Methodology:**

A retrospective review of electronic medical records (2016–2024) was conducted at a tertiary TB center in Qatar, targeting patients smear-positive at two months. Data included demographics, disease extent (e.g. cavitary lesions), initial and two-month acid-fast bacilli (AFB) smear counts, two-month AFB cultures, drug resistance, and comorbidities.

**Results:**

We identified 88 patients who remained smear-positive at two months of treatment. Among them, 61.4% were culture positive. Patients without cavitary lesions on the initial chest X-ray and those with two-month AFB counts < 10/100 fields had a 69% negative predictive value for culture negativity.

**Conclusions:**

Over half of persistent smear-positive patients remain potentially infectious at two months. However, those without cavitary lesions and with low AFB counts in the two months smear could be candidates for earlier isolation discontinuation, optimizing resources and reducing patient burden. These findings support individualized isolation protocols.

**Clinical trial number:**

Not applicable.

## Introduction

Pulmonary tuberculosis (TB) is a major global health challenge, with airborne transmission being the primary mode of spread. Respiratory isolation is a commonly used public health measure to reduce TB transmission, but guidelines for its duration and implementation vary [1]. The decline in smear positivity and infectivity following the initiation of effective treatment is well-documented, yet the timeline for patients to become noninfectious varies significantly. According to the CDCs Guidelines for Preventing the Transmission of Mycobacterium tuberculosis in Health-Care Settings, 2005, discharge before sputum smear conversion can be considered if the patient is clinically stable, on effective therapy with DOT arranged, has appropriate follow-up, no vulnerable household contacts, and agrees to remain home except for medical visits until they become smear negative. Patients with high-risk contacts should remain hospitalized under airborne isolation. However, because culture and drug-susceptibility results are not usually available when discontinuation decisions are made, the CDC recommends that hospitalized patients with TB remain under airborne precautions until they have received at least two weeks of effective multidrug therapy, shown clinical improvement, and produced three consecutive negative with AFB sputum smears collected 8–24 hours apart (with at least one being an early morning specimen [2]. Certain patients continue to test smear-positive for an extended time despite receiving effective treatment, necessitating prolonged isolation and the challenges that come with it. In some of these cases, smear positivity may only indicate the presence of non-viable bacilli or non-tuberculous mycobacteria, resulting in unnecessary isolation and a strain on resources. Prolonged isolation can have significant adverse effects on patients, including psychological distress, stigmatization, and socioeconomic challenges, particularly for marginalized populations [3, 4].

This study sought to determine the proportion of culture-positive cases among patients remaining smear-positive after two months of treatment and, since two-month culture results take several weeks, to identify factors predicting culture-negative status in these persistently smear-positive patients. By addressing these objectives, we hope to contribute to the development of more nuanced isolation guidelines that balance infection control with patient well-being and resource optimization.

## Methodology

A retrospective review of electronic medical records was conducted at the Communicable Disease Center (CDC), Hamad Medical Corporation (HMC)—Qatar’s principal national referral facility for tuberculosis care from 1/1/2016 to 28/2/2024. While TB diagnosis can occur at other primary or secondary health centers, all smear-positive pulmonary TB cases requiring inpatient respiratory isolation are referred to CDC-HMC as per national protocols. The CDC manages over 95% of the country’s inpatient TB isolation cases, particularly those needing prolonged isolation. The study included pulmonary TB patients who remained sputum smear-positive beyond two months of supervised, effective treatment under respiratory isolation. Data collected included demographics, disease extent (e.g., cavitary lesions on chest X-ray, disseminated disease), initial and two-month AFB smear counts, two-month AFB cultures and time to positivity, drug resistance profiles, comorbidities (e.g., diabetes, HIV, immunosuppression), and any treatment modifications.

The AFB smear microscopy and culture was performed at the National TB Reference Laboratory, HMC, Qatar, which is a College of American Pathologists (CAP)accredited laboratory. The heat fixed AFB smear was prepared from N-acetyl-L-cysteine–Sodium Hydroxide (NALC-NaOH) treated sputum specimens; followed by staining using Fluorochrome (Auramine O) method for screening and Ziehl–Neelsen (ZN) Method for confirmation of positive smears. Fluorescent-stained smears were screened using high-power fields (HPF) with fluorescence microscopy. ZN-stained smears were confirmed using oil immersion fields (OIF). Smear grading was performed as per WHO and CLSI guideline [5, 6]. The TB culture was conducted using the automated liquid culture BACTEC MGIT 960 system (BD, USA) following the manufacturer’s instructions. Positive MGIT culture tubes were further analyzed through visual inspection, smear microscopy of the culture media, and the TBcID assay (BD, USA) to identify and confirm the presence of the *Mycobacterium tuberculosis complex*. All DST procedures were conducted at the National TB Reference Laboratory, a CAP-accredited facility, in accordance with WHO-recommended protocols. TB treatment for drug susceptible and drug resistant TB were according to WHO guidelines [7]. Per Qatar’s National Tuberculosis Program, all suspected pulmonary TB cases undergo AFB smear, GeneXpert, and culture with drug susceptibility testing for rifampicin, isoniazid, ethambutol, and pyrazinamide. Smear-positive patients are placed in airborne isolation in isolation facility, and is discontinued only after two consecutive negative smears. Sputum cultures are routinely repeated at 2 and 6 months to monitor treatment response and detect resistance [8]. The overall TB incidence in Qatar is between 20 to 37 cases per 100,000 population [9].

The statistical analysis was designed to evaluate the relationship between persistent smear positivity and culture status at two months of treatment, as well as to identify predictors of culture negativity. Categorical variables were displayed as counts and percentages, whereas normally distributed continuous variables were summarized as means and standard deviations. Non-normally distributed variables were represented by the median, 25th, and 75th percentiles. The Shapiro-Wilk test and histograms were employed to check data for normality. The T-test was used for normally distributed variables, and the Mann-Whitney test for non-parametric variables. Categorical variables were evaluated using the Chi-square test. Univariable analysis was performed to identify the potential predictors of culture conversion in smear positive patients. To evaluate the independent effect of each factor, multivariable logistic regression was applied. A Receiver Operating Characteristic (ROC) curve was then plotted to determine the optimal cutoff point where sensitivity and specificity were maximized. Using this cutoff, we created a confusion matrix to assess the ability of the AFB count in smear and presence of cavity in predicting culture conversion. Based on the findings, the newly identified predictor was added to a multivariable logistic regression model, along with the presence of lung cavity in initial chest x-ray, to help refine the predictive model and enhance classification accuracy. Statistical significance was considered as a two-tailed p-value less than 0.05. Stata/SE 14.2 was used for analysis.

## Results

During the study period, 3,762 patients were diagnosed with pulmonary tuberculosis requiring isolation, of whom 88 patients with persistent smear positivity beyond two months of treatment were included in the analysis (Table 1). The median age was 41.5 years, and 84% were male. Mean AFB smear count in the initial smear was 650/100 fields, in chest x-ray, cavitary lesions were found in 67% (59/88), and bilateral or multi-lobar involvement in 84% (74/88). One patient had multidrug-resistant TB (MDR-TB), nine had isoniazid mono-resistance, and six had pyrazinamide mono-resistance. HIV co-infection was rare (1/88), while 32% (28/88) had uncontrolled diabetes.

**Table 1:** Baseline characteristics

Variable	All cohort (N = 88)	Smear positive & culture negative (N = 34)	Smear positive & culture positive (N = 54)	p-value
Age*	41.5 (31.5–49)	43.00 (36.00–50.00)	40.50 (30.00–48.00)	0.48
Male gender	74 (84%)	30 (88.24%)	44 (81.48%)	0.4
Multi drug resistant MTB	1 (1.14%)	0 (0.00%)	1 (1.85%)	0.42
Pyrazinamide mono resistance	6 (6.82%)	2 (5.88%)	4 (7.41%)	0.78
Isoniazid mono resistance	9 (10.23%)	2 (5.88%)	7 (12.96%)	0.29
Uncontrolled Diabetes mellitus	28 (31.82%)	12 (35.29%)	16 (29.63%)	0.58
HIV positive	1 (1.14%)	1 (2.94%)	0 (0.00%)	0.2
Cavity in the initial chest Xray	59 (67.05%)	16 (47.06%)	43 (79.63%)	0.002
Pleural effusion	4 (4.55%)	2 (5.88%)	2 (3.70%)	0.63
Disseminated TB	4 (4.55%)	1 (2.94%)	3 (5.56%)	0.57
Treatment modification due to drug induced liver injury or drug rash	10 (11.36%)	2 (5.88%)	8 (14.81%)	0.2
Number of AFB in initial smear*	650 (100–2000)	500 (100–1000)	900 (100–2500)	0.22
Days taken of initial culture positivity*	7.00 (6–9)	7.00 (5.00–11.00)	7.00 (6.00–9.00)	0.59
Number of AFB in 2 months smear*	10 (5–40)	5 (3–15)	20 (5–50)	< 0.001
days taken of 2 months culture positivity*	29 (22–36)	0	29 (22–36)	

* Nonparametric data presented as median (IQR), and n (%) for categorical measures

**Fig. 1:**
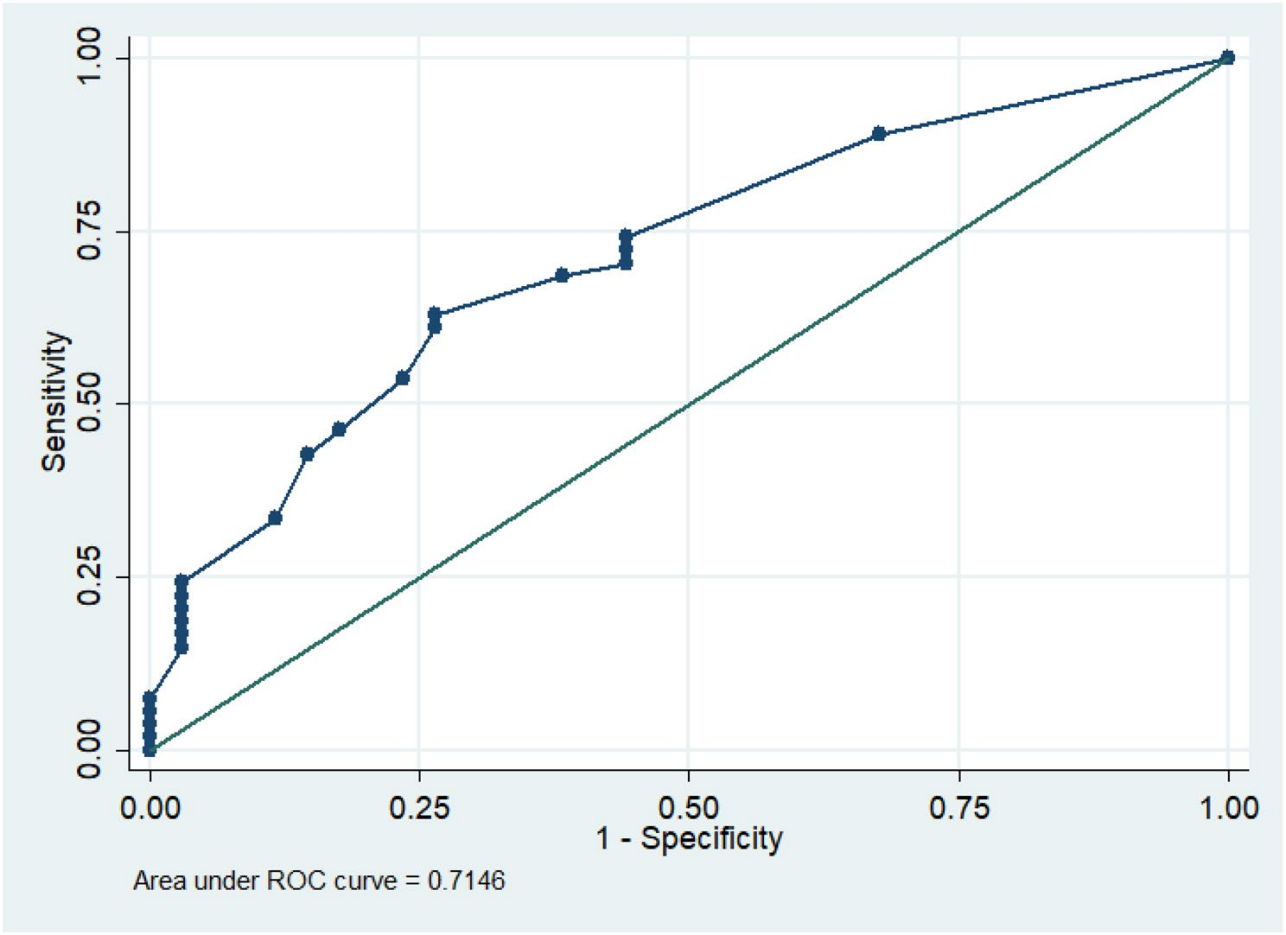
ROC curve at AFB count at 2 months

After two months of anti-TB treatment, 61.4% (54/88) of the cohort remained culture positive. Among patients who remained culture-positive at two months, the average time to culture positivity increased from 7 days before treatment to 29 days at the two-month mark, indicating reduced bacterial load Compared to culture-negative patients, those who remained culture-positive at two months had a significantly higher prevalence of cavitary lesions in initial chest x-ray (79% vs. 47%, *p* = 0.002) and higher AFB counts in two-month-smears (20/100 fields vs. 5/100 fields, *p* < 0.001). ROC curve analysis (Fig. 1) identified an AFB cutoff of 10/100 fields as predictive of two-month culture positivity (AUC: 71%, sensitivity: 63%, specificity: 74%). Patients without cavitary lesions and AFB < 10/100 fields had a negative predictive value (NPV) of 69% for two-month culture negativity (OR for culture conversion was 7.72, 95% CI: 1.90–31.36, *p* = 0.004) (Table 2).

**Table 2:** Multivariable logistic regression analysis of factors associated with two-month culture positivity

	Adjusted OR (95% Conf. interval)	P value
Diabetes mellitus	0.667 (0.213–2.088)	0.486
AFB > 10/100 field with presence of lung cavity	7.723(1.902–31.359)	0.004
Age	0.971(0.929–1.015)	0.189
Isoniazid monoresistance	4.332(0.692–27.138)	0.117
Pyrazinamide monoresistance	6.03(0.522–69.637)	0.15
Bilateral involvement in chest Xray	0.483(0.1471–1.584)	0.23
Drug induced hepatitis	2.116(0.332–13.507)	0.428
Days taken for initial culture positivity	0.861(0.729–1.017)	0.245

## Discussion

Our cohort comprised of advanced pulmonary disease, as indicated by extensive radiographic changes and high initial AFB counts in sputum smears. Despite a clear treatment response evidenced by a decline in AFB count, 61.4% of persistent smear-positive patients remained culture-positive at two months, indicating the presence of viable *M. tuberculosis*. This challenges the assumption that smear positivity during treatment solely reflects non-viable bacilli and highlights the need for a more thorough assessment of infectiousness.

Patients without cavitary lesions and with low AFB counts ( < 10/100 fields) at two months were more likely to be culture-negative (NPV = 69%), indicating a lower transmission risk and potential eligibility for earlier de-isolation. This supports evidence linking lower smear burden and limited radiographic disease with faster culture conversion. Our findings highlight the need to individualize isolation decisions in high-burden TB settings, balancing infection control with the harms of prolonged isolation.

Data from diverse experimental studies suggest that infectiousness rapidly declines after effective treatment initiation, However, the exact duration of treatment required to render people with TB disease non-infectious remains uncertain [4]. Cough aerosol studies and guinea pig models have shown that infectiousness declines rapidly after effective treatment, often before smear or culture conversion [10–14]. The notion that tuberculosis infectivity decreases after two weeks of treatment, with bacilli considered non-viable, was a postulation rather than an evidence-based conclusion [15]. Contrary to this, a prospective cohort study in Qatar evaluated culture conversion in smear-positive pulmonary tuberculosis patients after two weeks of treatment, finding that 95.7% remained culture-positive, suggesting persistent infectivity [16].

While the National Tuberculosis Coalition of America (NTCA) guidelines for respiratory Isolation and restrictions in community TB settings suggest that patients on effective anti-TB treatment for at least five days may be considered noninfectious or low-risk for transmission, regardless of sputum smear or culture results, they also recommend extended respiratory isolation in high-risk settings, such as those involving vulnerable groups like young children or immunosuppressed individuals, though the ideal isolation duration remains unclear [12]. The guidelines highlight that AFB smear and culture are poor predictors of infectiousness; effective therapy is the key determinant, and treatment interruption can restore infectiousness [17]. Canadian guidelines permit discontinuation of airborne precautions in persistently smear-positive patients after at least 4 weeks of effective therapy with clinical improvement [18]. In hot and humid Middle Eastern countries, where people often spend extended periods in air-conditioned spaces with recirculated air, these factors emphasize the need for refined isolation protocols that minimize unnecessary patient burden while ensuring effective infection control.

It can be argued that culture positivity confirms viable *M. tuberculosis* but does not fully capture infectiousness, which also depends on cough dynamics and aerosol generation [19, 20]. Newer tests, including cough aerosol culture, have been explored to predict treatment response and guide de-isolation. Studies show a rapid decrease in culture-positive MTB in cough aerosol on effective treatment [21–25]. However, it should be noted that recent studies showed that daily tidal breathing can generate higher bacillary count than cough per 24 hours [26].

Transcriptomic analysis of bacterial RNA released into the environment has been investigated as a way to study the decrease in infectiousness. Research indicates that within four days of starting treatment, bacterial mRNA levels decrease by more than 98%, indicating a swift decline in the number of viable bacilli [27]. Furthermore, transcriptional profiling shows a reduction in the expression of genes linked to virulence and pathogenicity, implying that *M. tuberculosis* becomes less capable of initiating new infections [28].

Since culture takes several weeks, a faster technique to assess bacterial viability is fluorescein diacetate vital staining. This technique identifies viable bacteria with preserved membrane integrity and has been proposed as a way to detect treatment failure before culture results are available [29, 30].

A limitation of this study is that, although culture positivity confirms viable *M. tuberculosis*, it does not directly imply active transmission. Animal model studies could help assess the transmissibility of these viable bacilli, potentially refining infection control protocols and treatment efficacy assessments. Furthermore, the modest sample size of 88 patients may restrict the generalizability of these findings to the broader tuberculosis population.

## Conclusion

In our study of advanced TB cases with persistent smear positivity beyond two months, we found that the majority remained culture-positive, indicating the presence of viable *M. tuberculosis* and a potential risk of transmission. While over half of persistent smear-positive patients remain culture-positive, a distinct subgroup (e.g., low AFB counts, no cavities) may be considered for earlier de-isolation under controlled settings. This supports a risk-stratified approach to isolation. The psychological adverse effects of prolonged isolation should be considered. Further research is necessary to refine our understanding of transmission risk and optimize isolation protocols.

## Data Availability

The datasets used and/or analyzed during the current study are available from the corresponding author on reasonable request.
